# The topology of evolutionary novelty and innovation in macroevolution

**DOI:** 10.1098/rstb.2016.0422

**Published:** 2017-10-23

**Authors:** Douglas H. Erwin

**Affiliations:** 1 Department of Paleobiology MRC-121, National Museum of Natural History, Smithsonian Institution, PO Box 37012, DC 20013-7012, USA; 2 Santa Fe Institute, 1399 Hyde Park Road, Santa Fe, NM 87501, USA

**Keywords:** morphospace, convergence, novelty, innovation, topology, adaptive landscape

## Abstract

Sewall Wright's fitness landscape introduced the concept of evolutionary spaces in 1932. George Gaylord Simpson modified this to an adaptive, phenotypic landscape in 1944 and since then evolutionary spaces have played an important role in evolutionary theory through fitness and adaptive landscapes, phenotypic and functional trait spaces, morphospaces and related concepts. Although the topology of such spaces is highly variable, from locally Euclidean to pre-topological, evolutionary change has often been interpreted as a search through a pre-existing space of possibilities, with novelty arising by accessing previously inaccessible or difficult to reach regions of a space. Here I discuss the nature of evolutionary novelty and innovation within the context of evolutionary spaces, and argue that the primacy of search as a conceptual metaphor ignores the generation of new spaces as well as other changes that have played important evolutionary roles.

This article is part of the themed issue ‘Process and pattern in innovations from cells to societies’.

## 1. Introduction

Wright introduced evolutionary spaces in 1932 [1] and the metaphor has been employed by evolutionary biologists ever since, with spaces representing an array of combinatoric possibilities, whether they are defined by genes or genomes, morphology, function or other traits. These spaces are generally visualized as scalar fields of multiple, independent biological variables with some having a dependent variable representing fitness, adaptation, complexity or performance. Adaptive landscapes have been widely employed to describe the trajectories of lineages while morphospaces have been employed by paleontologists seeking to understand the distribution of form within clades. Waddington's introduction of the epigenetic landscape extended Wright's metaphor to development [2]. Evolutionary spaces have also been adopted in social sciences, including economics [3]. In some cases, the relationship between spaces has received considerable attention, as with genotype–phenotype mapping in small RNAs [4,5] and in empirical studies [6,7]. In other cases, the relationship between different spaces is not well established. Many aspects of evolutionary spaces have been recently reviewed [8–12] and Dawkins devoted a book to the subject [13].

Spaces have played a role in conceptualizing evolutionary novelty since the work of Simpson, however, recent insights from comparative developmental biology and from the fossil record suggest a need to re-examine the relationship between spaces and novelty. This contribution addresses the relationship between these spaces of combinatoric possibility and evolutionary novelty and innovation, and particularly how the topology (or inferred topology) of spaces may influence evolutionary dynamics. Evolutionary novelty has been described as a process of search for combinations of attributes that increase fitness. This perspective assumes that the spaces exist rather than being constructed by organisms, that search is a meaningful driver of novelty, and that novelty occurs within a pre-defined space rather than by extensions to the space or the generation of new spaces. There are substantial reasons for doubting each of these assumptions. This paper considers the topology of novelty and innovation, some of the ways in which evolutionary spaces may evolve as a consequence of novelty, and the relative importance of search versus construction in generating novelty and innovation.

## 2. Novelty versus innovation

The terms novelty, innovation and, in technology, invention are often used interchangeably and without clear definitions. This is unfortunate, as a critical question in evolutionary biology is whether novelty differs from traditional processes of adaptation [14–17]. Following Schumpeter [18], I distinguish novelty (invention for Schumpeter) from innovation [14]. Comparative developmental studies of animals have revealed unexpectedly deep homologies in genes and mechanisms and established that adaptive evolution is not necessarily the sole path to the developmental changes that generate phenotypic novelties [15,19,20]. Novelty reflects the formation of newly individuated characters, features of the organism which were not present in ancestral species. This definition has emerged from a considerable body of study among biologists [15–17,21,22]. Novel characters in turn may have many different character states: a feather is a novel character but the many different sorts of feathers represent alternative character states, but not novelties [15], just as a new gene may have many different alleles. This definition is broad enough to encompass novelties at other levels, for example, the origin of new regulatory mechanisms or behavioural patterns, as well as novel morphologies. From this conceptual framework, major evolutionary transitions involving the formation of new evolutionary individuals [23], such as the eukaryotic cell, are extreme cases of novelties.

Mayr [24] and Simpson [25] viewed novelties as a new structure with a new function responding to ecological opportunities, as with Simpson's discussions of adaptive radiations [26]. Thus, any suggestion of a disconnect between the origin of novelties and their success would likely have struck them as odd, or even bizarre. But evidence from fossils shows that new morphological features often arise long before they become ecologically or evolutionarily significant (macroevolutionary lags) [14]. For example, grasses originated and diversified into their major clades at least 25 million years before grasslands became widespread [27]. In this case the presence of phytoliths (silica bodies unique to grasses) in sediments during the lag interval demonstrate that grasses were present, but were ecologically insignificant. These lags are not cases of failure of the fossil record, but instances where novelty has occurred but with little ecological impact (since palaeontologists commonly track taxic diversity rather than ecological abundance, the number of cases of macroevolutionary lags is probably underestimated). Innovation occurs when a novelty has sufficient ecological or evolutionary impact that removing the node represented by that taxa from an ecological network (not simply a food web) would noticeably impact the structure or functioning of the network. In some cases novelties may coincide with innovation, but lags show that novelty does not necessarily lead immediately to innovation. Further support for this distinction between novelty and innovation comes from quantitative studies of morphospaces, which have shown that novelties that define a new clade generally define the boundaries of the morphospace, with morphologic disparity exceeding taxonomic diversity. Subsequent adaptive evolution fills in the space [9,28]. Although these definitions differ from those adopted in this collection [29] and elsewhere (e.g. [30]), they are consistent with an ongoing research program.

## 3. Evolutionary spaces: a bestiary

In 1879 Lewis Carroll published ‘A new Puzzle’ in which two words had to be linked by a string of other words, each differing by a single letter [31] (nonsense four-letter words were excluded). Carroll used the example of head to tail through head–heal–teal–tell–tall–tail. Since the length of the word is fixed and a single letter changes at each step, the puzzle defines a space of 26^4^ or 456 979 possible four-letter strings. Wright's genotype spaces use the same logic as Carroll's puzzle. Wright examined networks of genotypes or gene combinations as a tool to convey his quantitative work to a broader biological audience (although Provine noted that over his career Wright used fitness spaces in three mutually incompatible ways [32]). Wright's network of gene combinations represents a gene or genotype space, but the addition of fitness of different gene combinations as a dependent variable converted the space into a surface or landscape. Dobzhansky [33] immediately extended Wright's approach by envisioning multiple niches within a single space [10]. Simpson converted fitness landscapes into adaptive landscapes [34], deploying them in support of his view of the relationships between microevolutionary process and macroevolutionary patterns, including the formation of new higher taxa through invasion of new adaptive zones. Despite critiques [35], the Wright, Dobzhansky and Simpsonian landscapes have proven quite generative (see papers in [8]).

Initial work on two- and three-dimensional landscapes assumed that selection would drive populations to adaptive peaks, although in landscapes with multiple peaks populations might find themselves stranded on local optima with no path to higher peaks. Wright recognized that actual landscapes were highly multi-dimensional, and later work suggested that multi-dimensionality turns peaks and valleys into large flat surfaces, punctuated with holes with very low fitness and that populations might often move along ridges in multi-dimensional space [36]. The impact of epistatic effects was examined in the N–K model, which generalized Wright's models and showed that high degrees of epistasis produce very rugged landscapes and led to the description of novelty as the ‘adjacent possible’ [37,38]. Research on empirical landscapes has explored how their topologies influence the course of evolution [39,40].

Maynard Smith recalled Carroll's puzzle when he described a protein space in which the letters represent different amino acids [41]. He employed the protein space model to illustrate how one gene can change into another and suggested that functional proteins were connected by a path consisting of single base pair changes, each of which produced a functional protein. Thus, embedded within the protein space were networks of functional proteins. Maynard Smith's straightforward protein space was extended to the space of folded structures that can be generated from repeats of the helix–loop–helix–loop motif [42]; known repeat proteins occupy only a small part of the space.

The peaks in these spaces represent high fitness or adaptation, but Waddington inverted the model in his epigenetic landscapes to show differentiating cells falling into basins of attraction. This was part of his critique of an over-reliance on fitness in the Modern Synthesis [2,43,44]. Waddington's landscape was highly influential, particularly in studies of cellular differentiation (e.g. [45]) and led to a variety of developmental spaces [46–48]. Spaces of gene regulatory networks (GRNs), the mechanistic basis of development, have been described as a regulatory space, as discussed further below.

Although the idea of a space of morphologies was inherent in Simpson's work, quantitative analysis of morphology was equally influenced by the pioneering grid deformations of D'Arcy Thompson [49–51]. These were not fully articulated until Raup's work on logarithmically coiled organisms [52,53] used three coiling parameters to define a combinatoric space of morphologic possibilities. Raup investigated what part of the space had been occupied by molluscs and brachiopods. One useful feature of this theoretical morphospace was that it allows examination of morphologies that do not exist. Empirical morphospaces are defined by measurements of fossil or living taxa [54,55]. Projection of phylogenetic trees into morphospaces (to produce a ‘phylomorphospace’) revealed how clades evolved within a morphospace over time. Although morphospaces can be used for closely related taxa, they are most commonly employed by paleontologists studying larger, but morphologically similar clades; the need for some similar (homologous) characters limits the morphologic breadth which can be studied.

The metaphor of spaces has been applied in other biological contexts, not always explicitly connected to genotype spaces. Examples include a skeletal design space used to explore the variety of skeletal elements used in different animal clades [56,57]; the range of ecological strategies adopted by animals in an ecospace [58]; and spaces have been employed to examine the relationship between phenotype and function, whether at the biochemical level [59] or organismal phenotype [60]. Changes in disparity associated with Phanerozoic mass extinctions defined an extinction space to study the morphological impact of different events [61]. Not all of the spaces described here are also landscapes, either because no fitness value is included (morphospaces, ecospace, some trait spaces), or because the entities are discontinuous and so no landscape is possible (e.g. skeleton space) (table 1). Some of these are special cases of a phenotypic space, and some might argue that all should reduce to a genotype space. As discussed below, emergent features in developmental, morphologic, ecologic and functional spaces inhibit mapping these to genotype space.

**Table 1.** Features of evolutionary spaces. Protein spaces have been viewed as the mapping from a sequence space, but in a broader context both protein and developmental spaces are intermediaries between genetic and phenotypic spaces, with novelties and innovations largely occurring in different types of spaces Most of the spaces discussed here are variants of phenotypic spaces. Wagner-reg and Wagner-met are the regulatory and metabolic spaces described in Wagner [62]. In general, morphospaces are not landscapes, but see McGhee [11] for a discussion of morphospaces as adaptive landscapes. ‘Variable’ indicates that topology of the space or involvement in novelty or innovation may vary depending on the taxonomic breadth under study.

evolutionary space	space or landscape	topology	novelty or innovation
genotype—Wright	both	metric	novelty
genotype—NK	both	metric	novelty
protein	both	metric	novelty
developmental	both	variable	novelty
Wagner-reg	space	Euclidean	novelty
Wagner-met	space	Euclidean	novelty
adaptive (Simpsonian)	landscape	variable	variable
morphospaces			
landmark	space	locally Euclidean grading to pre-topology with increasing morphologic scope	variable
no landmarks	space	topology to pre-topology	variable
skeletal design	space	set	novelty
phenotypic trait	variable	variable	innovation
functional	variable	variable	innovation
ecospace	space	set	innovation

Evolutionary spaces have been deployed in other contexts. For example, fitness landscapes and particularly the Kauffman–Levin N–K landscapes spread to the social sciences (reviewed in [3,63]). Raup's theoretical morphospace and general principles of network architecture were used to generate a network morphospace spanning food webs to neural and electrical circuits [64]. This space was used to examine common network properties and to evaluate unexplored architectures. In development economics, a product space shows the products produced in different countries and has been used to argue that economic development is driven by moving into products adjacent to those already produced in a country [65].

## 4. Topology

The folding of small RNA molecules has provided a powerful tool for understanding evolutionary topology, and particularly the relationship between the genotype (the RNA sequence) and the three-dimensional structure produced by folding of the RNA sequence (the phenotype). Only a few two-dimensional structures will have the lowest possible free energy, so mapping an RNA sequence into a two-dimensional structure is relatively straightforward. In this model a sequence 100 nucleotides long will be adjacent to 300 other sequences which could be reached by a single-nucleotide change so the total sequence space is 10^60^. Since many changes to a sequence will not generate a change in the folded structures, there will be large networks of effectively neutral changes. Single base-pair changes to the RNA sequence may trace a path through the sequence space, but single base-pair changes can also produce very different optimal two-dimensional structures. If different two-dimensional structures are taken as novelties the critical point is that some random networks will have many adjacencies to other random networks, leading to the expectation that shifting between the two folded structures represented by different networks should be relatively easy (think of the boundary between France and Germany—a step out of France is likely to land in Germany). By contrast, other networks will be relatively isolated, with few adjacencies to other neutral networks (Monaco is surrounded by France, but few steps out of France would land in Monaco; the converse is not true, as any step out of Monaco necessarily lands in France) [4]. The novel structures represented by the isolated networks will be relatively inaccessible, but for this example it is the topology of the sequence space that determines the accessibility of the phenotypic novelties.

Thus, accessibility is not necessarily the same as distance (the distance may be short but if the probability of the change is low the new form has low accessibility). Studies of this RNA sequence model have shown that there is not necessarily an easy path through sequence space between any two phenotypes [4,5,66], but that alternative shapes may be only a few mutations away in any sequence. Wagner extended the concept of genotype spaces to gene regulation with a regulatory genotype space containing the set of all possible circuit topologies of a given size [62,67]. Two circuits are neighbours in such a space if they differ in one regulatory interaction. As with many spaces, many regulatory circuits are expected to produce the same pattern of gene expression, although single changes might produce novel regulatory patterns. This is a microevolutionary model for novelty, driven by search through a sequence space (or its equivalent).

The concept of distance turns out to be critical in evaluating the topology of evolutionary spaces. We live in a world of three spatial and one time dimension, with the spatial dimensions being regular and symmetric. Such Euclidean spaces are special cases of metric spaces, a vector space where the dimensions of the space are orthogonal and distances can be computed among all elements. The RNA folding space is not Euclidean because while adjacent neutral networks may be in the same neighbourhood, no meaningful distance can be computed between them, just as no meaningful distance can be computed between a mushroom and a coffee cup. Non-Euclidean spaces range from metric spaces where distances can be measured but dimensions may not be orthogonal, to topological spaces where objects may be near or adjacent, but distances cannot be quantified. In pre-topological spaces objects may be in a neighbourhood, but the space is unbounded (in contrast to the bounded Euclidean, metric and topological spaces). For RNA the sequence space is Euclidean but the two-dimensional phenotype space is a pre-topological space. A set is an unbounded space where even the concept of a neighbourhood does not apply. We tend to apply ‘Euclidean intuitions’ [68] without inquiring into whether the space is, in fact, Euclidean. For example, Raup's shell coiling model that introduced morphospaces is non-Euclidean [69]. Moreover, apparent distances computed in morphospaces may be misleading where constraints on variation limit the range of possible morphotypes [70]. The evolutionary spaces described above include a range of topologies (table 1), although many spaces are often assumed to be at least metric, if not Euclidean [4,5,68,71]. A substantial challenge in evolutionary biology, and one that has achieved too little attention, is adjusting our Euclidean intuitions to the evolutionary dynamics in spaces of different topologies.

## 5. Topology of evolution

The enduring interest in evolutionary spaces reflects the fact that while some spaces are a description of empirical patterns, all of the spaces provide useful intuitions about the evolutionary process (which is why Wright introduced genotypic spaces). This section addresses the relationships and possible discontinuities between spaces, and then elaborates on the difference between adaptive searches within an existing space and the construction of new spaces.

One might argue that all spaces are ultimately derived from a vast sequence space and mappings from that into a phenotypic space. But the existence of discontinuities between spaces is implicit in Wagner's distinction between macromolecular, regulatory and metabolic spaces. Two factors reveal the extent of discontinuities: the RNA folding model only includes two-dimensional structures because the three-dimensional folding is not predictable. In general, mapping from sequence space to other spaces will be limited by alternative minimal energy configurations and the intercession of accessory proteins and other factors. Furthermore, for multicellular forms phenotypes reflect cellular, mechanical and environmental inputs [72] as well as sequence. Environmental factors may have particularly important feedbacks in developmental processes. Genome sequencing has shown that variations in gene number or genome size are largely unrelated to developmental or phenotypic complexity. Since roughly the same number of genes (18 000 to 20 000) is needed to make any animal, the complexity of animals reflects not variation in sequence space but in the regulatory interactions that enable development. Thus, the various spaces are not necessarily decomposable to a sequence space. Wright seems to have recognized this in distinguishing between genetic (sequence) and genotypic spaces. While sequence is a critical input to phenotypic spaces, sequence is increasingly disconnected as one progresses down the list of spaces in table 1.

In the RNA folding model the sequence space is defined by the length of the sequence, movement through the space is via single base changes and the phenotype (the folded state) is predictable from the sequence space. As noted, search in the Euclidean sequence space can produce novel forms in the phenotypic pre-topology space. The RNA model has been extended to regulatory and metabolic networks [62,73]. A combinatoric space of regulatory circuits defines a regulatory space, with neighbouring circuits differing by one interaction, as with the spaces described earlier. The distance in such a space would be the number of differences in non-zero regulatory interactions, and Wagner has argued that as with the RNA genotype space, many regulatory circuits would produce the same gene expression pattern, forming a vast network of viable regulatory circuits (and that the same principles apply to metabolic networks). Thus sequence, protein and Wagner's regulatory spaces are defined *a priori* and exist in what Wagner describes as ‘…the timeless eternal realm of nature's libraries’ [74, p. 176]. Novelty arises through single mutations moving a circuit from one phenotype to an adjacent but novel phenotype; in other words, search through a network.

Empirical examples of search through evolutionary spaces include functional analysis of a variety of labrid fish jaw structures to show that they are equivalent, despite their morphological variety [60]. The diverse topologies of myogenic GRNs across bilaterians [75] demonstrate that substantial changes in GRN architecture can occur while generating the same outcome (a phenomenon known as developmental systems drift [76]). Both cases illustrate the many to one mapping between genotype and phenotype spaces (broadly defined), and that novelties at one level do not necessarily generate novelties at another level. Evolution via search in a metric or Euclidean space can lead to novelty in the relevant phenotypic space, but is more likely to lead to adaptive change rather than novelty as defined previously.

The evolutionary operator in these models is a single-nucleotide or amino acid change, but even in microbial systems this is far too limited. In principle, evolutionary spaces could change in three ways in addition to search or diffusion: growth or extension of existing spaces via new axes, the generation of new operators for diffusion within an existing space and the construction of new spaces. Real sequence spaces are not the Hamming spaces (of equal length) envisioned in the RNA model, but of variable length due to nucleotide deletions, insertions and frame-shift mutations, new genes may be assembled by formation of new domains or rearrangement of existing domains, and regulatory spaces can change via addition or deletion of transcription factor binding sites. Such changes will impact the dimensionality of these vast spaces. New evolutionary operators change patterns of search within a space, potentially changing the accessibility of potential novelties. Examples of new operators include alternative splicing and horizontal gene transfer. Surveys of protein evolution have established the importance of many factors beyond those captured by a protein space, such as the position of the gene within the genome and pleiotropic effects such as a protein's position within a biological network or its dispensability [77].

Evolutionary novelties and innovations have also resulted in the construction of new evolutionary spaces. New spaces involve a combination of characters which are partly or wholly non-homologous with characters in other spaces. This may be most obvious in phenotypic spaces where unique character combinations define distinctive morphologies. The appearance of such new spaces may be abrupt, as with the origin of icthyosaus during the early Triassic (about 250 million years ago (Ma)), or gradual, as with the appearance of early birds during the Mesozoic. Cognitive expansion in social learning also appears to involve the construction of new spaces [78].

The origin of animals illustrates the limitations of relying on a search rather than a construction-based view of novelty and innovation. The closest living relatives of Metazoa are choanoflagellates and then, slightly more distant, filastreans. Molecular clock studies indicate that the origin of Metazoa occurred approximately 780 Ma although animals do not appear in the fossil record until approximately 560 Ma with the Cambrian Explosion of animal life beginning after 541 Ma [79,80]*.* Although some specific genes are related, animal genomes are about two times larger than their ancestors; many proteins include novel domains or arrangements of domains [81]; regulatory networks have evolved through the construction of new types of circuits [19,82]; and the range of operators that change regulatory interactions has expanded, as with co-option of subcircuits within a GRN. Developmental spaces are discontinuous because of the introduction of the means of cellular coordination, the introduction of distal enhancers near the origin of Metazoa [83], and the generation of entirely novel developmental spaces via signalling pathways and microRNAs. These novelties complicate the mapping from genotype through development to phenotype. Although it is true that these reside in the DNA sequence, their activities are emergent phenomena that depend upon interactions with other regulatory elements, various proteins and environmental factors. The phenotypic or morphospaces that emerged during the Cambrian Explosion are highly discontinuous [79] and, despite highly conserved genes, the developmental processes leading to trilobites or crinoids are vastly different. This is not to say that we cannot calculate a phylogenetic distance between animals and their ancestors; that is easily done with molecular, genomic or morphologic data. But such phylogenetic distance estimates largely represent acquisition of new shared, derived characters, many of them novelties, associated with the origins of metazoan clades.

Evolutionary spaces have provided a powerful and enduring metaphor for exploring evolutionary dynamics. However, the application of these spaces to novelty and innovation has been hampered by a failure to distinguish novelty from innovation, and a reliance upon search through existing spaces as the dominant metaphor. Empirical evidence as well as theoretical considerations suggest that evolutionary spaces have evolved through the construction of novelties and innovations. Acknowledging the importance of the extension, modification and generation of new spaces as a key component of novelty does not diminish the importance of search. Rather, search involves the exploration of viable combinatoric solutions once a space has been generated. Thus, any examination of the evolutionary topology of innovation must necessarily address the macroevolutionary dimension of construction of new spaces as well as the microevolutionary aspects of search. The approach I advocate here is similar to a recent simulation of increases in cultural tools. This model identified four different types of innovation, including ‘main-branch tools’ that construct a niche for the associated expansion of toolkits, and recombination of existing tools [84]. Although that study did not explicitly invoke evolutionary spaces, the main-branch tools represent the generation of a novel space, while the toolkit innovations are a search through the space (with the interesting caveat that there is often an order in which new tools may be acquired, as is also the case in biology [85]).

## 6. Discussion

The nature of evolutionary spaces is relevant for considerations of novelty and innovation for three reasons. First, while if one considers similar morphologies the appropriate phenotype space may be (locally) Euclidean, as the scope of the morphologies expands the nature of the space becomes progressively less defined until even the concept of a neighbourhood disappears (in mathematical terms, large phenotypic spaces are manifolds). Second, while some evolutionary spaces, particularly sequence spaces, exist *a priori*, others are constructed through evolution, thus spaces evolve, not just the entities which occupy them. This leads to the third point: although novelty and innovation have been described as search or diffusion through evolutionary spaces, they also generate new evolutionary spaces. Thus, a critical question for any consideration of the topology of evolution is: Do different types of novelties and innovations occur in spaces with different topologies? Or, to put it a different way: Do different types of spaces allow, or facilitate, different types of novelties or innovation?

Some types of spaces, particularly phenotypic, morphometric, trait and functional spaces, may be available but ecologically or environmentally precluded. In other words, particular traits or states are genetically or developmentally accessible but cannot survive (and thus would not be observed). Separating novelty from innovation acknowledges that the former may occur in these spaces without necessarily leading to sufficient ecological impact to generate innovation. Conversely, extinction, particularly mass extinctions, have eliminated many developmental, phenotypic, trait and functional spaces, and morphospaces through the loss of genetic and developmental potential, as with the disappearance of trilobites [86]. Whether this has simply rendered the spaces inaccessible or truly caused their disappearance is an interesting issue for future work.

The ubiquity of phenotypic convergence between phylogenetically independent lineages has led some authors to offer this as evidence for the limited potential of evolution [11,87,88]. In biology, culture and technology there are many examples of multiple origins of the same or similar traits: beavers evolved once in the time of dinosaurs [89] and again more recently, complex states evolved multiple times [90] and there are many examples of repeated evolution in science and technology [91]. Experimental evolutionary studies of microbes [92,93] and protein sequences of fish anti-freeze [94] exhibit similar patterns of convergent evolution (see review of biological convergence in [95]). As McGhee put it ‘…the number of evolutionary pathways available to life is not endless, but is instead quite limited’ [95, p. 94], which has contributed arguments over the relative importance of contingency and determinism in evolution [96,97]. McGhee's work has examined adaptive and morphospaces in particular, although I know of no systematic comparison of the scope of convergence across different evolutionary spaces. Some convergence reflects physical requirements, as with the similarities between tuna, dolphins and some marine reptiles. But the limitations on evolution may be more apparent than real, reflecting search within an existing space, rather than the generation of new spaces.

Where the genotype to phenotype mapping is straightforward, as in the case of the RNA example discussed several times, search is an appropriate approach to understanding the discovery of novel functions. In cases where the mapping is more complex, as with development in multicellular organisms or in cultural and technological domains, understanding how the introduction of new operators or the generation of new spaces occurs may be a more fruitful approach. These considerations suggest that evolvability generated by novelties may be closely related to the ability to generate these new operators or new spaces.

Evolution as the cumulative effect of small genetic changes is the essence of microevolution. The classic model of search through genotypic and phenotypic spaces, whether driven by selection or drift through neutral networks, reflects such a viewpoint. In contrast, the generation of new operators as well as the generation of new evolutionary spaces reflects macroevolutionary change. Much of the macroevolutionary theory generated by palaeontologists over the past few decades focuses on changing distributions of species and clades and documentation of discontinuities between micro- and macroevolution [98–100]. The wealth of new comparative information on developmental mechanisms has generated renewed interest in distinct, macroevolutionary sources of variation [15,19,101,102].

## 7. Future directions

The metaphor of evolutionary spaces has been widely adopted across biology and has penetrated other fields. Despite some well-founded criticisms this metaphor seems unlikely to disappear. Evolutionary landscapes provide insights into patterns of evolutionary transition at different scales and may be particularly useful in developing models of novelty and innovation. Evolutionary novelties, as defined here, may be associated with search or extension via new axes of existing spaces, the generation of new operators for diffusion and the construction of new spaces. This framework raises questions for further work: Can we express the expansion of developmental regulatory controls for animals in a developmental space? Are there specific cases where both the construction or expansion of spaces can be examined along with search through a space? Do different types of novelties or innovations arise when new spaces are constructed versus those that arise from search? Since the framework developed here rejects claims that spaces are universal, how have spaces changed over time, whether within a clade or more generally? Does the topology of a space influence the types of evolutionary change that can occur? These questions suggest that examinations of evolutionary topology may provide intriguing insights into novelty and innovation, not just in biology but across other domains as well.

## Data accessibility

This article has no additional data.
